# One novel representation of DNA sequence based on the global and local position information

**DOI:** 10.1038/s41598-018-26005-3

**Published:** 2018-05-15

**Authors:** Zhiyi Mo, Wen Zhu, Yi Sun, Qilin Xiang, Ming Zheng, Min Chen, Zejun Li

**Affiliations:** 1 School of Information and Electronic Engineering, Wuzhou University, Wuzhu, China; 2 College of Computer Science and Electronic Engineering, Hunan University, Hunan, China; 3 College of Computer and Information Science, Hunan Institute of Technology, Hengyang, China

## Abstract

One novel representation of DNA sequence combining the global and local position information of the original sequence has been proposed to distinguish the different species. First, for the sufficient exploitation of global information, one graphical representation of DNA sequence has been formulated according to the curve of Fermat spiral. Then, for the consideration of local characteristics of DNA sequence, attaching each point in the curve of Fermat spiral with the related mass has been applied based on the relationships of neighboring four nucleotides. In this paper, the normalized moments of inertia of the curve of Fermat spiral which composed by the points with mass has been calculated as the numerical description of the corresponding DNA sequence on the first exons of beta-global genes. Choosing the Euclidean distance as the measurement of the numerical descriptions, the similarity between species has shown the performance of proposed method.

## Introduction

The graphical and numerical representation of DNA, RNA or protein sequences has become the popular strategies to analyze the evolutionary relationship between species. As the availability of varies gene data for different species, the comparison of different organisms that own unique genetic information involves in mathematics, biology, physics, informatics and so on. Many researchers have focused on the issue of representation of gene sequence, as seen in^1–31^, so the study of representation of gene sequence is significant and beneficial.

Hamori and Ruskin^32^ first proposed the H-curve, the graphical representation of nucleotide sequence, which is convenient for the visual analysis and comprehension of the DNA sequences. Following them, further researches of representation of DNA sequence were carried^33–44^. For example, Zhang^45^ proposed a five-color map visualization of DNA sequences named ColorSquare. Jafarzadeh^1^ constructed the C-curve with no loss of information. And Aram^5^ introduced a new graphical representation of the DNA sequences which called spider representation. Moreover, Bielinska-Waz^10^ represented the sequence with a set of discrete lines which referred to as the B−spectrum. Unfortunately, owing to the high degeneracy and loss of information and the need of a lot of space in the transformation of DNA sequence to graphical representation, the performances of many methods are not satisfactory as expected.

To solve those problems, we present one novel representation of DNA sequence based on global and local position information. Distinct from previous reports, the more effective representation is obtained and the possible effect caused by different length of DNA sequence is restrained by new method. In detail, the novel concept of representation of DNA sequence involves (1) formulating the graphical representation of DNA sequence according to the curve of Fermat spiral which remaining the global position information of the original sequence, (2) taking the local position information of DNA sequence into consideration according to attach each point in the curve of Fermat spiral with the related mass, (3) the normalized moments of inertia of the curve of Fermat spiral which composed by the points with mass has been calculated as the description of the corresponding DNA sequence on the first exons of beta-global genes.

## Graphical representation of DNA sequence

In order to make full use of global information of DNA sequence, the original DNA sequence is divided into four subsequences constituted by A, C, G or T that four point sets correspondingly can be obtained by the position of nucleotide in the original DNA sequence. Thus, each nucleotide in the subsequence corresponds to one point in the set. With the operation by distributing each point set to the curve of Fermat spiral, four corresponding curves which means the graphical representation of DNA sequence can be plotted. The reason that we choose the Fermat spiral instead of the circle as the distribution curve of subsequence is that the curve of Fermat spiral is the monotonically increasing functions in the polar coordinate system which can remaining the information of position of the original sequence.

We regard the DNA sequence as BS(base sequence) which is constituted by four subsequences of AS, CS, GS and TS. Concretely, the i-th nucleotide in BS is denoted as V_i_^BS^, i = 1, 2, ···, N_BS_. It is obvious that the length of nucleotide in base sequence is equal to the total length of nucleotide in four subsequence, described as:

1 $${N}_{BS}={N}_{AS}+{N}_{CS}+{N}_{GS}+{N}_{TS}$$

where N_BS_, N_AS_, N_CS_, N_GS_ and N_TS_ respectively denote the length of nucleotide in base, A, C, G and T subsequence. For the purpose of plotting the base curve of Fermat spiral corresponding to the base sequence, the coordinate of points in the polar coordinate system are calculated according to the information of position in the base sequence. For each point, calculated as:

2 $${\theta }_{{V}_{i}^{BS}}=\frac{2\pi \,}{(L-1)}\times ({L}_{{V}_{i}^{BS}}-1)$$

where ${{\rm{\theta }}}_{{{\rm{V}}}_{{\rm{i}}}^{{\rm{BS}}}}$ denotes the polar angle of nucleotide ${{\rm{V}}}_{{\rm{i}}}^{{\rm{BS}}}$ in the polar coordinate system; L is one constant which means the shortest length of DNA sequence for different species in the experience; ${L}_{{{\rm{V}}}_{{\rm{i}}}^{{\rm{BS}}}}$ denotes the position of nucleotide ${{\rm{V}}}_{{\rm{i}}}^{{\rm{BS}}}$ in the base sequence which ranging from 1 to ${{\rm{N}}}_{{\rm{BS}}}$. The mathematical formula of the curve of Fermat spiral is described as:

3 $${\rho }_{{V}_{i}^{BS}}=\sqrt{\,{\theta }_{{V}_{i}^{BS}}}\,$$

As for the nucleotides in the base sequence, the corresponding set of coordinate for each point in the polar coordinates are calculated as

$$\{{{\rm{p}}}_{{{\rm{V}}}_{1}^{{\rm{BS}}}}({{\rm{\theta }}}_{{{\rm{V}}}_{1}^{{\rm{BS}}}},{{\rm{\rho }}}_{{{\rm{V}}}_{1}^{{\rm{BS}}}}),{{\rm{p}}}_{{{\rm{V}}}_{2}^{{\rm{BS}}}}({{\rm{\theta }}}_{{{\rm{V}}}_{2}^{{\rm{BS}}}},{{\rm{\rho }}}_{{{\rm{V}}}_{2}^{{\rm{BS}}}}),\,\ldots ,\,{p}_{{{\rm{V}}}_{{\rm{i}}}^{{\rm{BS}}}}({{\rm{\theta }}}_{{{\rm{V}}}_{{\rm{i}}}^{{\rm{BS}}}},{{\rm{\rho }}}_{{{\rm{V}}}_{{\rm{i}}}^{{\rm{BS}}}}),\,\ldots ,\,{{\rm{p}}}_{{{\rm{V}}}_{{{\rm{N}}}_{{\rm{BS}}}}^{{\rm{BS}}}}({{\rm{\theta }}}_{{{\rm{V}}}_{{{\rm{N}}}_{{\rm{BS}}}}^{{\rm{BS}}}},{{\rm{\rho }}}_{{{\rm{V}}}_{{{\rm{N}}}_{{\rm{BS}}}}^{{\rm{BS}}}})\}.$$

**Figure 1:**
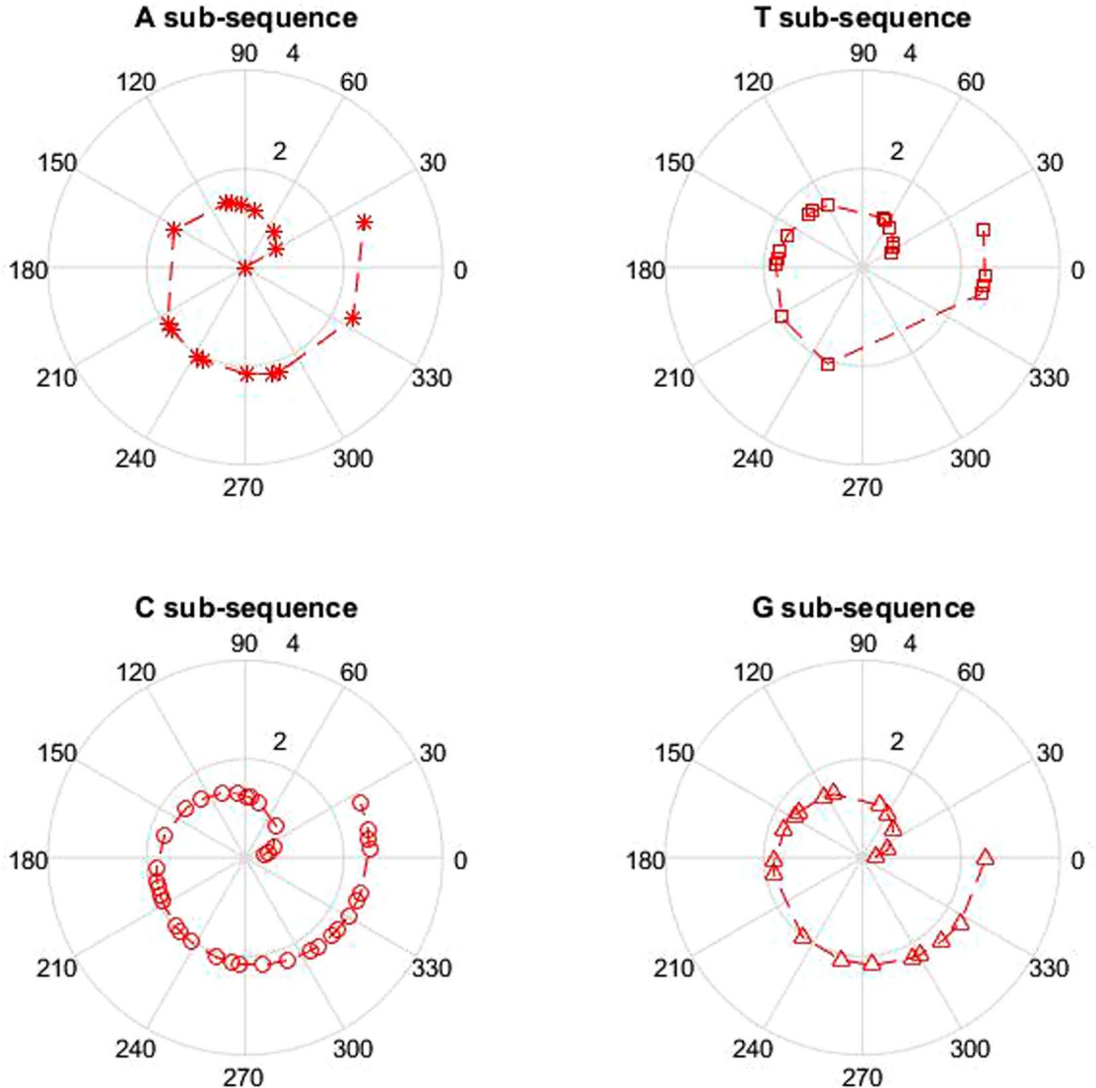
The graphical representation of human gene. From left to right and from top to bottom, the graphical representations are respectively for A, C, G and T subsequence.

Correspondingly, four subsets can be obtained and plotted. As shown in Fig. 1, the graphical representation of the first exons of β-globin gene of human DNA gene is plotted.

## Attaching each point with a mass

In order to make full use of carried information of DNA sequence, the local characteristics are taken into consideration to attach each point corresponding to the nucleotide in the base sequence with a mass. Since one of immediate 5′ neighbor nucleotide and two of immediate 3′ neighbor nucleotides were considered as the context to calculate the mass of point corresponding to the second nucleotide in the group, the times and the compactness that the second nucleotide occurs and arranges are considered as the criterion to confirm the mass of the second nucleotide in the group.

According to the times that the nucleotide same as the second position repeats in the group, four categories may be divided. As shown in the following the nucleotide being same as the second nucleotide is denoted as ***1*** and the nucleotide being different from the second nucleotide is denoted as ***0****.*

1. ***0100***
2. ***0101, 1100, 0110***
3. ***1101, 1110, 0111***
4. ***1111***

For example, the nucleotide of second position occurs one time in the first category. And according to the analysis of the four categories, six situations are obtained by the compactness that the second nucleotide arranges. For example, as for the second category that the nucleotide of second position occurs two times, its first situation of ***0101*** in which the two ***1*** are separated by on***e 0*** and its second situation in which the two ***1*** are compactly arranged.

1. ***0100***
2. ***0101***
3. ***1100, 0110***
4. ***1101***
5. ***1110, 0111***
6. ***1111***

Therefore, the different mass in $\{\frac{1}{6},\frac{2}{6},\frac{3}{6},\frac{4}{6},\frac{5}{6},1\}$ is attached to the point corresponding to the nucleotide of second position. However, for the purpose of reducing the impact of DNA sequence which is too long, the mass of latter sequence after ***L*** are restrained as4where  denotes the mass of the point corresponding to the nucleotide of ${{\rm{V}}}_{{\rm{i}}}^{{\rm{BS}}}$ after restraint; *ε* denotes the scale of constraint which is one constant of ***0.0375*** in experiment. So the points corresponding to the positions later than ***L*** own bigger polar radius but smaller mass; on the one hand, this characteristic can restrain the difference on length of dissimilar species; on other, it also can reserve the smaller difference on length of similar species.

## Numerical Representation

For the widespread application of the moment of inertia in many gene numerical representation method^10,11,15,16^, the normalized moments of inertia for each massive sub-curve of Fermat spiral are calculated as the numerical representation of formal DNA sequence in this paper. To the convenience of calculation, the transformation of polar coordinates to plane coordinates is performed:

5 $$\{\begin{array}{c}{x}_{{V}_{i}^{BS}}={\rho }_{{V}_{i}^{BS}}\times cos\,{\theta }_{{V}_{i}^{BS}}\\ {y}_{{V}_{i}^{BS}}={\rho }_{{V}_{i}^{BS}}\times sin\,{\theta }_{{V}_{i}^{BS}}\end{array}$$

Since the point ${{\rm{p}}}_{{{\rm{V}}}_{{\rm{i}}}^{{\rm{BS}}}}({{\rm{\theta }}}_{{{\rm{V}}}_{{\rm{i}}}^{{\rm{BS}}}},{{\rm{\rho }}}_{{{\rm{V}}}_{{\rm{i}}}^{{\rm{BS}}}})$ in the polar coordinates is transformed to point ${{\rm{p}}}_{{{\rm{V}}}_{{\rm{i}}}^{{\rm{BS}}}}({{\rm{x}}}_{{{\rm{V}}}_{{\rm{i}}}^{{\rm{BS}}}},{y}_{{{\rm{V}}}_{{\rm{i}}}^{{\rm{BS}}}})$ in the plane coordinates, the center of mass for the massive curve of Fermat spiral in the plane coordinates system is calculated as:

6 $$\{\begin{array}{c}\tilde{{x}_{{V}^{BS}}}=\frac{1}{{N}_{BS}}\sum _{i=1}^{{N}_{BS}}{m}_{{V}_{i}^{BS}}\times {x}_{{V}_{i}^{BS}}\\ \,\tilde{{y}_{{V}^{BS}}}=\frac{1}{{N}_{BS}}\sum _{i=1}^{{N}_{BS}}{m}_{{V}_{i}^{BS}}\times {y}_{{V}_{i}^{BS}}\end{array}$$

So the ordinate of the center of mass is point $\tilde{{{\rm{p}}}_{{{\rm{V}}}^{{\rm{BS}}}}}(\tilde{{{\rm{x}}}_{{{\rm{V}}}^{{\rm{BS}}}}},\,\tilde{{{\rm{y}}}_{{{\rm{V}}}^{{\rm{BS}}}}})$, the moment of inertia of the massive curve is described as:

7 $${M}_{BS}={\sum }_{i=1}^{{N}_{BS}}{m}_{{V}_{i}^{BS}}\times distance({p}_{{V}_{i}^{BS}},\tilde{{p}_{{V}^{BS}}})$$

where distance $({{\rm{p}}}_{{{\rm{V}}}_{{\rm{i}}}^{{\rm{BS}}}},\tilde{{{\rm{p}}}_{{{\rm{V}}}^{{\rm{BS}}}}})$ denotes the squared distance, calculated as:

8 $$distance({p}_{{V}_{i}^{BS}},\tilde{{p}_{{V}^{BS}}})={({x}_{{V}_{i}^{BS}}-\tilde{{x}_{{V}_{i}^{BS}}})}^{2}+{({y}_{{V}_{i}^{BS}}-\tilde{{y}_{{V}_{i}^{BS}}})}^{2}$$

*The normalized moment of inertia* is described as:

9 $${r}_{BS}=\sqrt{\frac{{M}_{BS}}{{\sum }_{i=1}^{{N}_{BS}}{m}_{{V}_{i}^{BS}}}}$$

There ${r}_{BS}$, one 4-dimensional vector ${r}_{BS}=[{r}_{AS},{r}_{CS},{r}_{GS},{r}_{TS}]$, denotes the numerical representation of DNA sequence consisted of A, T, C and G subsequences. Following, the similarity distance between species is calculated according to the Euclidean measurement:

10 $$S(\alpha ,\beta )={[{\sum }^{}{|{{\rm{r}}}_{{\rm{BS}}}^{{\rm{\alpha }}}-{{\rm{r}}}_{{\rm{BS}}}^{{\rm{\beta }}}|}^{2}]}^{\frac{1}{2}}$$

where ${{\rm{r}}}_{{\rm{BS}}}^{{\rm{\alpha }}}$ and ${{\rm{r}}}_{{\rm{BS}}}^{{\rm{\beta }}}$ respectively denote the numerical representations of species α and β. So *S*(*α*, *β*) denotes the similarity distance between vectors ${{\rm{r}}}_{{\rm{BS}}}^{{\rm{\alpha }}}$ and ${{\rm{r}}}_{{\rm{BS}}}^{{\rm{\beta }}}$ in the 4-dimensional space.

## Results and Discussion

We test the performance of proposed method in the standard dataset that popular in the field of the DNA representation research, as seen in Table 1, the first exons of β-globin gene of different species. According to Eq. (9), Table 2 shows the numerical representations of DNA sequence for each target species. After obtaining the numerical representation consisted of 4-dimensional vectors, Table 3 shows the similarity/dissimilarity between pairs of species according the description of Eq. (10).

**Table 1:** The first exons of β-globin gene of different species.

k	Species	Gene ID	N
1	Human	U01317	92
2	Gorilla	X61109	93
3	Chimpanzee	X02345	105
4	Rat	X06701	92
5	Mouse	V00722	93
6	Lemur	M15734	92
7	Rabbit	V00882	92
8	Goat	M15387	86
9	Bovine	X00376	86
10	Opossum	J03643	92
11	Gallus	V00409	92

**Table 2:** The numerical representation of DNA sequence.

Species	*r*_*AS*_	*r*_*CS*_	*r*_*GS*_	*r*_*TS*_
Human	1.6674	1.7921	1.7233	1.7689
Gorilla	1.6674	1.7921	1.7233	1.7727
Chimpanzee	1.6885	1.8085	1.7239	1.7845
Rat	1.6074	1.7858	1.8242	1.7462
Mouse	1.5943	1.7480	1.8407	1.7921
Lemur	1.6781	1.6659	1.8149	1.8046
Rabbit	1.6454	1.7145	1.8690	1.7809
Goat	1.5416	1.6808	1.8246	1.8476
Bovine	1.5416	1.5929	1.8056	1.8471
Opossum	1.5693	1.6713	1.9416	1.7398
Gallus	1.7986	1.6879	1.9639	1.7140

**Table 3:** Similarity/dissimilarity matrix under the Euclidean distance.

Species	Human	Gorilla	Chimp	Rat	Mouse	Lemur	Rabbit	Goat	Bovine	Opossum	Gallus
Human	0	0.0038	0.0309	0.1198	0.1470	0.1604	0.1670	0.2114	0.2616	0.2696	0.2983
Gorilla	0.0038	0	0.0292	0.1206	0.1465	0.1596	0.1668	0.2100	0.2604	0.2701	0.2991
Chimp	0.0309	0.0292	0	0.1365	0.1620	0.1707	0.1782	0.2281	0.2804	0.2870	0.2988
Rat	0.1198	0.1206	0.1365	0	0.0631	0.1513	0.0987	0.1602	0.2282	0.1684	0.2583
Mouse	0.1470	0.1465	0.1620	0.0631	0	0.1208	0.0683	0.1031	0.1763	0.1393	0.2582
Lemur	0.1604	0.1596	0.1707	0.1513	0.1208	0	0.0832	0.1443	0.1608	0.1792	0.2131
Rabbit	0.1670	0.1668	0.1782	0.0987	0.0683	0.0832	0	0.1355	0.1844	0.1209	0.1940
Goat	0.2114	0.2100	0.2281	0.1602	0.1031	0.1443	0.1355	0	0.0900	0.1618	0.3215
Bovine	0.2616	0.2604	0.2804	0.2282	0.1763	0.1608	0.1844	0.0900	0	0.1921	0.3433
Opossum	0.2696	0.2701	0.2870	0.1684	0.1393	0.1792	0.1209	0.1618	0.1921	0	0.2324
Gallus	0.2983	0.2991	0.2988	0.2583	0.2582	0.2131	0.1940	0.3215	0.3433	0.2324	0

For the comparison, Table 4 shows the similarity/dissimilarity between Human and other species in some other methods similarly taking the Euclidean distance as the measurement. From Table 4, finding that most listed methods^1,2,10,46,47^ also make the same conclusion that Gorilla are the most similar species to Human and Chimp is the next similar species to Human except method^33^ which make the similar conclusion that Chimp is the most similar species to Human and Gorilla is the next similar species to Human. Besides, some listed methods^2,10,47^ also make the same conclusion that Gallus is the most dissimilar species to Human.

**Table 4:** Similarity/dissimilarity between Human and other species with different methods.

Methods	Gorilla	Chimp	Rat	Mouse	Lemur	Rabbit	Goat	Bovine	Opossum	Gallus
Our work	0.0038	0.0309	0.1198	0.1470	0.1604	0.1670	0.2114	0.2616	0.2696	0.2983
Randic *et al*. 2003^33^	0.0210	0.0170	0.0430	0.0830	0.0870	0.0420	0.0610	0.0840	0.1480	0.1090
Dai *et al*. 2006^46^	0.0120	0.0155	0.0704	0.0543	0.0603	0.0287	0.0169	0.0276	0.1389	0.1146
Liu and Wang 2006^47^	0.3070	0.3101	0.4256	0.3089	0.3688	0.2968	0.4341	0.4172	0.3805	0.4479
Liao *et al*. 2013^2^	0.1651	0.4688	0.9202	0.6024	1.0110	0.7453	0.6010	0.6320	1.3710	1.5932
Jafarzadeh *et al*. 2013^1^	0.0330	0.0920	0.2160	0.1630	0.1940	0.1240	0.1650	0.2210	0.1940	0.1940
Bielinska-Waz *et al*. 2017^10^	0.0056	0.0314	0.1838	0.2395	0.2497	0.1844	0.1276	0.0872	0.3904	0.4687

**Figure 2:**
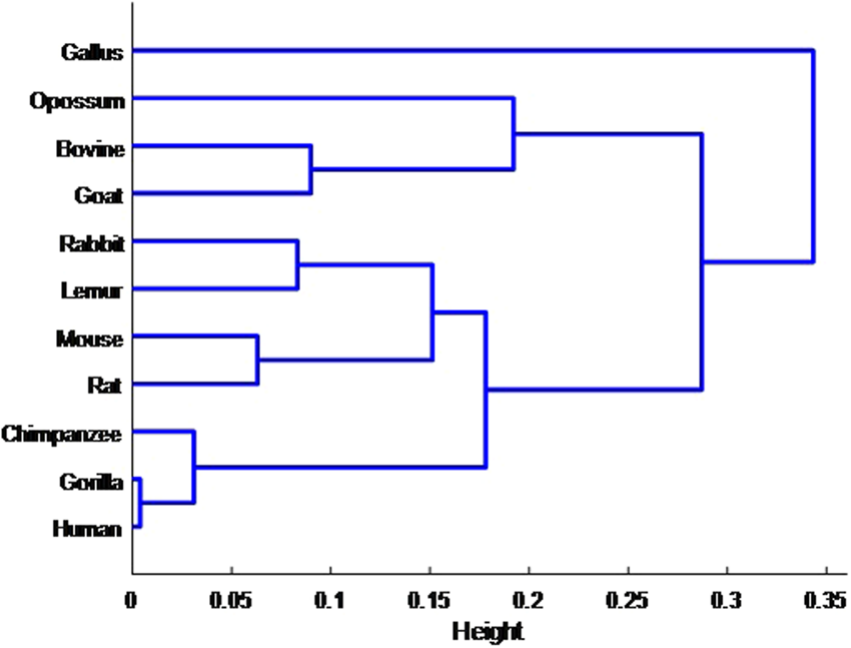
Cluster dendrogram.

Two most distinct dendrogram corresponding to the Euclidean measures is plotted in Fig. 2. As seen, the similar cluster pairs are respectively as Human-Gorilla(same cluster result in^1,2,10,40,47,48^), Rat-Mouse(same result in^10,49^), Lemur-Rabbit(same cluster result in^10^), Goat-Bovine(same cluster result in^10,33,40,47–49^), Human-Gorilla-Chimpanzee (same cluster result in^1,10,33,40,46,48,49^).

**Figure 3:**
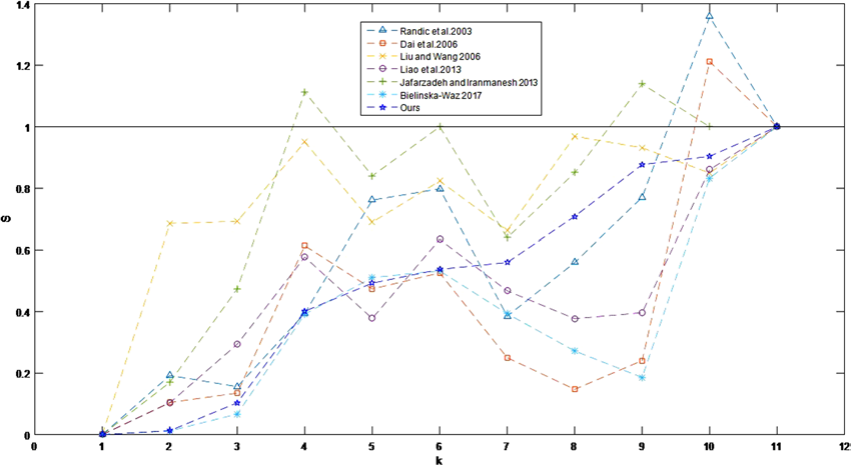
Similarity values of human-other species with different methods.

Normalizing S^human−gallus^ = 1 to the convenience of the visualization for results in other paper^1,2,10,33,46,47^ which similarly using the Euclidean measurement. As shown in Fig. 3, different methods perform different results that may be useful with different consideration.

In conclusion, the paper presents a novel method to extract the characteristic of the DNA sequence with the graphical and numerical operations which can effectively achieve the similarity/dissimilarity comparison of different species. In this method, the distribution of sequence to the curve of Fermat spiral remains the global position information successfully and the attachment of the mass to the point remains the local position information successfully. Specifically in our result, the group of Rat-Mouse- Lemur- Rabbit is more similar to the group of Human- Gorilla-Chimpanzee compared with the group of Goat- Bovine-Opossum which may be helpful to the exploration of the evolutionary relationship between species. Moreover, the similar pairs that obtained by our method illustrate the performance of proposed representation of DNA sequence.
